# Evidence for positive selection acting on microcystin synthetase adenylation domains in three cyanobacterial genera

**DOI:** 10.1186/1471-2148-8-256

**Published:** 2008-09-22

**Authors:** Ave Tooming-Klunderud, David P Fewer, Thomas Rohrlack, Jouni Jokela, Leo Rouhiainen, Kaarina Sivonen, Tom Kristensen, Kjetill S Jakobsen

**Affiliations:** 1 University of Oslo, Department of Molecular Biosciences, 0316 Oslo, Norway; 2 University of Oslo, Department of Biology, Centre for Ecological and Evolutionary Synthesis (CEES), 0316 Oslo, Norway; 3 University of Helsinki, Department of Applied Chemistry and Microbiology, P.O. Box 56, Viikki Biocenter, Viikinkaari 9, FIN-00014, Finland; 4 NIVA, Norwegian Institute for Water Research, 0411 Oslo, Norway; 5 Department of Biology, Microbial Evolution Research Group (MERG), University of Oslo, 0316 Oslo, Norway

## Abstract

**Background:**

Cyanobacteria produce a wealth of secondary metabolites, including the group of small cyclic heptapeptide hepatotoxins that constitutes the microcystin family. The enzyme complex that directs the biosynthesis of microcystin is encoded in a single large gene cluster (*mcy*). *mcy *genes have a widespread distribution among cyanobacteria and are likely to have an ancient origin. The notable diversity within some of the Mcy modules is generated through various recombination events including horizontal gene transfer.

**Results:**

A comparative analysis of the adenylation domains from the first module of McyB (McyB1) and McyC in the microcystin synthetase complex was performed on a large number of microcystin-producing strains from the *Anabaena*, *Microcystis *and *Planktothrix *genera. We found no decisive evidence for recombination between strains from different genera. However, we detected frequent recombination events in the *mcyB *and *mcyC *genes between strains within the same genus. Frequent interdomain recombination events were also observed between *mcyB *and *mcyC *sequences in *Anabaena *and *Microcystis*. Recombination and mutation rate ratios suggest that the diversification of *mcyB *and *mcyC *genes is driven by recombination events as well as point mutations in all three genera. Sequence analysis suggests that generally the adenylation domains of the first domain of McyB and McyC are under purifying selection. However, we found clear evidence for positive selection acting on a number of amino acid residues within these adenylation domains. These include residues important for active site selectivity of the adenylation domain, strongly suggesting selection for novel microcystin variants.

**Conclusion:**

We provide the first clear evidence for positive selection acting on amino acid residues involved directly in the recognition and activation of amino acids incorporated into microcystin, indicating that the microcystin complement of a given strain may influence the ability of a particular strain to interact with its environment.

## Background

Cyanobacteria produce a wealth of bioactive peptide derivatives with a broad range of biological activities and pharmacological properties [1]. Many of these are synthesized on nonribosomal peptide synthetases (NRPS). These megaenzyme complexes typically have a modular architecture. A typical module contains specific functional domains for activation, thioesterification, and condensation of amino acids [2]. Additional domains for the modification of activated amino acids such as epimerization, heterocyclisation, oxidation, formylation, reduction or *N*-methylation may also be present [2]. NRPS gene clusters in some cyanobacteria can occupy up to 5 percent of the genome [1].

The modular design of NRPS gene clusters promotes homologous recombination, including recombination within a gene cluster and intragenomic recombination between different gene clusters within the same genome or intergenomic recombination with DNA introduced from other cyanobacteria [3-5]. The cellular consequences of recombination will depend on several factors, including the phenotypic effects, if any, of the introduced DNA segment. In order to be successful, the new gene variant should at least not be detrimental to the host. For NRPS systems, important factors will be whether the novel peptide can fulfil the biological role(s) of the original peptide or provide new benefits to the host. Nonetheless, recombination within and among NRPS gene clusters potentially could constitute a mechanism for continuous alteration of the synthetases and peptide products.

**Figure 1:**
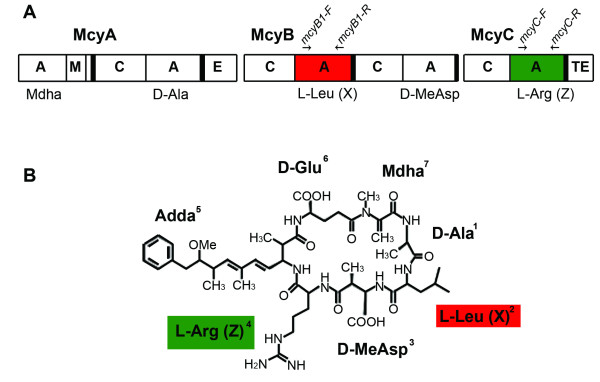
**Organization of the *mcyABC *gene cluster (A).** Adenylation domains investigated in the present study are indicated in red and green. The relative positions of primers (arrows) are shown. Genus-specific *mcyB *and *mcyC *primers are listed in Table 8. (B) The structure of microcystin-LR. Amino acid residues activated by the adenylation domains of McyB1 and McyC are indicated by red and green, respectively. Mdha is N-methyl-dehydroalanine, D-MeAsp is 3-methyl-aspartic acid and Adda is 3-amino-9-methoxy-2,6,8,-trimethyl-10-phenyl-4,6-decandienoic acid.

Among cyanobacterial NRPSs, the microcystin synthetase gene clusters (*mcy*) have been extensively studied. Microcystins are cyclic heptapeptides with common structure cyclo-D-Ala^1^-X^2^-D-MeAsp^3^-Z^4^-Adda^5^-D-Glu^6^-Mdha^7 ^where D-MeAsp is D-*erythro*-β-methyl-aspartic acid, Adda is 3-amino-9-methoxy-2,6,8-trimethyl-10-phenyl-(4*E*), (6*E*)-decadienoic acid, D-Glu is D-*iso*-glutamic acid, and X and Z are variable L-amino acids (Figure 1). Complete gene cluster sequences are available from strains within the *Anabaena*, *Microcystis *and *Planktothrix *genera [6-10]. Recombination in the *mcy *gene clusters has been reported to involve equivalent modules, i.e. modules with the same position in similar gene clusters [11-13], but also modules in different positions in similar gene clusters or from different gene clusters [3,14,15]. Although most strains can produce a range of microcystin isoforms there is a single *mcy *gene cluster in the genome [6-8,10,16,17], indicating that recombination events involving equivalent domains must be intergenomic.

The substrate specificity of the adenylation (A) domain is considered to be the primary determinant of substrate selection (for a review, see [18]). Recombination events involving A domains might lead to changes in substrate specificity and subsequently in the microcystin profile [3,15]. The A domains of modules McyB1 (the first module of the McyB protein) and McyC recognise and activate the amino acids that are incorporated in the variable positions X and Z of microcystin (Figure 1). These A domains have been extensively studied within *Microcystis *[13,15], and *Planktothrix *[11,14]. Within *Microcystis*, recombination has lead to the presence of two types of A domains in different strains [15]: a mainly Leu-activating A domain and a mainly Arg-activating A domain that has a high sequence similarity to the Arg-activating A domain in McyC (in the following, these two types of A domains in McyB1 are called B-type and C-like, respectively). Recombination involving A domain coding regions of *mcyB1 *and *mcyC *has been detected in *Microcystis *[13,15] and *Anabaena *[3,9].

Here, we compare McyB1 and McyC A domains in strains from the three main microcystin-producing genera: *Anabaena*, *Microcystis *and *Planktothrix *to investigate the role of genomic processes in the reshaping of microcystin genes, enzyme complexes and corresponding peptides. We have looked for signs of recombination, both between equivalent and non-equivalent modules, as well as mutations and selective forces acting on these A domains.

## Results

In the present study, we have compared A domains of microcystin synthetase modules McyB1 and McyC from altogether 58 strains including 21 *Anabaena*, 19 *Microcystis *and 18 *Planktothrix *strains with characterized microcystin-isoform profiles, including two non-producers (Table 1). The profiles made it possible to identify the amino acid residue(s) incorporated by each of these modules.

**Table 1:** Strains compared in present study

Strain^*#*^	Geographic origin	Year	Genes^*&*^	Microcystin isoforms produced [reference]	
					
			mcyB* mcyC		
***Anabaena***					
N-C 83/1	L. Edlandsvatnet, Norway	1981	EU009900 EU009918	[D-Asp^3^]MC-LR, [D-Asp^3^]MC-RR, MC-LR, MC-RR	[56]
N-C 267/4	L. Fammestadtjønni, Norway	1990	EU009901 EU009919	MC-HtyR, MC-LR, MC-FR, [D-Asp^3^]MC-LR, [D-Asp^3^]MC-HtyR, [D-Asp^3^]MC-FR, MC-HilR, MC-HphR	[56]
N-C 269/2	L. Frøylandsvatnet, Norway	1990	EU009902 EU009920	[D-Asp^3^]MC-LR, MC-HtyR, [D-Asp^3^]MC-HtyR, MC-LR, [D-Asp^3^]MC-FR, MC-FR, MC-HilR, MC-HphR, [D-Asp^3^]MC-HilR, [D-Asp^3^]MC-HphR	[56]
N-C 269/6	L. Frøylandsvatnet, Norway	1990	EU009903 EU009921	[D-Asp^3^]MC-LR, MC-HtyR, [D-Asp^3^]MC-HtyR, MC-LR, [D-Asp^3^]MC-FR, MC-FR, MC-HilR, MC-HphR, [D-Asp^3^]MC-HilR, [D-Asp^3^]MC-HphR	[56]
N-C 270/1	L. Arefjordsvatnet, Norway	1990	EU009904 EU009933	[D-Asp^3^]MC-LR, MC-LR, [D-Asp^3^]MC-RR, MC-RR	[56]
90	L. Vesijärvi, Finland	1986	AJ536156 AJ536156	MC-LR, [D-Asp^3^]MC-LR, MC-RR, [D-Asp^3^]MC-RR, MC-HilR, [D-Asp^3^]MC-HilR	[9]
1TU44S16	L. Tuusulanjärvi, Finland	2001	EU009887 EU009905	[D-Asp^3^]MC-LR, MC-LR	[56]
1TU30S4	L. Tuusulanjärvi, Finland	2001	EU009888 EU009906	[Dha^7^]MC-LR, [D-Asp^3^, Dha^7^]MC-LR, [L-Ser^7^]MC-LR	[56]
1TU31S9	L. Tuusulanjärvi, Finland	2001	EU009889 EU009907	[Dha^7^]MC-LR, [D-Asp^3^, Dha^7^]MC-LR, [L-Ser^7^]MC-LR, [D-Asp^3^, demet-N^7^]MC	[56]
202A1/35	L. Vesijärvi, Finland	1987	EU009890 EU009908	[D-Asp^3^, Dha^7^]MC-LR, [Dha^7^]MC-LR, [L-Ser^7^]MC-LR	[56]
1TU46S11	L. Tuusulanjärvi, Finland	2001	EU009891 EU009909	[D-Asp^3^]MC-LR, MC-LR, [D-Asp^3^]MC-HilR	[56]
202A2/41	L. Vesijärvi, Finland	1987	EU009892 EU009910	[D-Asp^3^, Dha^7^]MC-LR, [Dha^7^]MC-LR, [L-Ser^7^]MC-LR, [D-Asp^3^, demet-N^7^]MC	[56]
0TU33S16	L. Tuusulanjärvi, Finland	2000	EU009893 EU009911	[D-Asp^3^]MC-LR, MC-LR, [D-Asp^3^]MC-HilR	[56]
258	L. Tuusulanjärvi, Finland	1990	EU009894 EU009912	MC-LR, [D-Asp^3^]MC-LR, MC-HilR, [D-Asp^3^]MC-HilR	[56]
1TU32S11	L. Tuusulanjärvi, Finland	2001	EU009895 EU009913	[Dha^7^]MC-LR, [D-Asp^3^, Dha^7^]MC-LR, [L-Ser^7^]MC-LR	[56]
BIR 246	Gulf of Finland, Baltic Sea	2004	EU009896 EU009914	[D-Asp^3^]MC-HtyR, MC-HtyR, [D-Asp^3^]MC-LR, MC-LR, [D-Asp^3^]MC-FR, MC-FR, MC-HphR, [D-Asp^3^]MC-HphR, MC-HilR, D-Asp^3^]MC-HilR	[56]
288	Littoisten vesilaitos, Finland	1990	EU009897 EU009915	MC-HtyR, MC-LR, MC-FR, [D-Asp^3^]MC-LR, [D-Asp^3^]MC-HtyR, MC-HphR, [D-Asp^3^]MC-FR,	[56]
315	Gulf of Finland, Baltic Sea	1997	EU009898 EU009916	[Dha^7^]MC-HtyR, [D-Asp^3^, Dha^7^]MC-HtyR, [Dha^7^]MC-LR	[56]
318	Gulf of Finland, Baltic Sea	1998	EU009899 EU009917	MC-HtyR, [D-Asp^3^]MC-Hty, [D-Asp^3^]MC-LR, MC-LR	[56]
66A	L. Sääskjärvi, Finland	1986	EU151874 EU151867	[Dha^7^]MC-HtyR, [D-Asp^3^, Dha^7^]MC-HtyR, [Dha^7^]MC-HphR, [Dha^7^]MC-LR, [L-Ser^7^]MC-HtyR	[56]
18B	L. Vaaranlampi, Finland	1986	EU151873 EU151866	[D-Asp^3 ^Dha^7^]MC-RR, [Dha^7^]MC-RR	[56]

***Microcystis***					
N-C 31	Little Rideau Lake, Canada	1954	EU009866 *B* EF115396	MC-LR	[15]
N-C 57	L. Frøylandsvatnet, Norway	1978	EU009867 *C* EF115397	[Asp^3^, Dha^7^]MC-RR, [Dha^7^]MC-RR	[15]
N-C 118/2	L. Gjersjøen, Norway	1983	EU009868 *B* EF115398	[Asp^3^]MC-LR, MC-LR	[15]
N-C 140	Bendig's Pond, Canada	1975	EU009869 *B* EU009881	MC-LR, MC-desmethyl-LR	This work
N-C 143	L. Akersvatnet, Norway	1984	EU009870 *C* EF115399	None	[15]
N-C 160/2	L. Akersvatnet, Norway	1985	EU009871 *C* EU009882	None	This work
N-C 161/1	L. Mosvatnet, Norway	1985	EU009872 *B* EF115400	MC-YR, MC-LR	[15]
N-C 169/7	L. Arresø, Denmark	1985	EU009873 *C* EF115401	MC-RR, MC-LR	[15]
N-C 171/10	L. Arresø, Denmark	1985	EU009874 *C* EU009883	MC-LR, MC-YR, MC-RR	This work
N-C 228/1	L. Akersvatnet, Norway	1985	EU009875 *C* EF115402	[Dha^7^]MC-RR, [Dha^7^]MC-LR	[15]
N-C 264	L. Frøylandsvatnet, Norway	1990	EU009876 *C* EF115403	[Dha^7^]MC-RR	[15]
N-C 324/1	L. Tøråssjøen, Norway	1993	EU009877 *C* EF115404	[Asp^3^, Dha^7^]MC-RR, [Dha^7^]MC-RR, [Dha^7^]MC-LR, MC-LR	[15]
N-C 357	River Zala, Hungary	1996	EU009878 *C* EU009884	MC-RR, MC-LR, MC-YR, MC-desmethyl-LR	This work
N-C 496	Queen Elizabeth Channel, Uganda	2004	EU009879 *C* EU009885	MC-YR, MC-desmethyl-YR	This work
AB2002/24	Pilsner Pond, Kenya	2002	EU009880 *B* EU009886	MC-LR, desmethyl-MC-YR, MC-YR	[57]
UV027	Germany	ND	AF458094 *C* AF458094	MC-RR	[9]
PCC 7806	Braakman Reservoir, The Netherlands	1972	AF183408 *B* AF183408	MC-LR, [Asp^3^]MC-LR	[10]
K-139	Lake Kasumigaura, Japan	1985	AB019578 *B* AB019578	[Dha^7^]MC-LR, [Asp^3^, Dha^7^]MC-LR	[58]
NIES 102	Lake Kasumigaura, Japan	1982	AB092807 *C*	MC-LR, MC-RR, MC-YR	[58]

***Planktothrix***					
3	L. Mondsee, Austria	2001	AJ749276 AJ749285	[Asp^3^, Mdha^7^]MC-RR	[14]
64	L. Wörthersee, Austria	2001	AJ749277 AJ749286	[Asp^3^, Mdha^7^]MC-RR	[14]
111	L. Mondsee, Austria	2001	AJ749282 AJ749291	[Asp^3^, Mdha^7^]MC-RR	[14]
31/1	L. Wannsee, Germany	2001	AJ749267 AJ749294	[Asp^3^, Mdha^7^]MC-RR, [Asp^3^]MC-HtyR, [Asp^3^]MC-LR	[14]
32	L. Wannsee, Germany	2001	AJ749268 AJ749295	[Asp^3^, Mdha^7^]MC-RR, [Asp^3^]MC-LR	[14]
39	L. Wannsee, Germany	2001	AJ749269 AJ749296	[Asp^3^, Mdha^7^]MC-RR, [Asp^3^]MC-LR	[14]
79	L. Arresø, Denmark	2001	AJ749270 AJ749297	[Asp^3^, Mdha^7^]MC-RR, [Asp^3^]MC-LR	[14]
SAG 6.89	L. Plußsee, Plön, Germany	1969	AJ749271 AJ749298	[Asp^3^, Mdha^7^]MC-RR, [Asp^3^]MC-LR	[14]
N-C 126/8	L. Langsjön Finland	1984	AJ441056 AJ441056	[Asp^3^, Mdha^7^]MC-RR, [Asp^3^]MC-LR	[6]
80	L. Schwarzensee, Austria	2001	AJ749278 AJ749287	MC-HtyR	[11]
82	L. Ammersee, Germany	2001	AJ749279 AJ749288	[Asp^3^, Dhb^7^]MC-RR, [Asp^3^]MC-HtyR, [Asp^3^]MC-LR	[14]
108	L. Irrsee, Austria	2001	AJ749281 AJ749290	[Asp^3^, Dhb^7^]MC-RR, [Asp^3^]MC-LR	[14]
PCC 7821	L. Gjersjøen, Norway	1971	AJ749283 AJ749292	[Asp^3^, Dhb^7^]MC-RR, [Asp^3^]MC-LR	[14]
CCAP 1459/30	L. Plöner See, Germany	ND	AJ749284 AJ749293	[Asp^3^, Dhb^7^]MC-RR, [Asp^3^]MC-LR	[14]
CCAP 1459/11A	L. Windermere, UK	1975	AJ749272 AJ749299	[Asp^3^, Dhb^7^]MC-RR	[14]
CCAP 1459/21	Esthwaite Water, UK	1985	AJ749274 AJ749301	[Asp^3^, Dhb^7^]MC-RR	[14]
CCAP 1460/5	L. Kasumigaura, Japan	1983	AJ749275 AJ749302	[Asp^3^]MC-HtyR, [Asp^3^]MC-LR	[14]
CCAP 1459/16	Blelham Tarn, UK	1979	AJ749273 AJ749300	[Asp^3^]MC-HtyR, [Asp^3^]MC-LR	[14]

^*# *^N-C, NIVA-CYA, Norwegian Institute for Water Research Cyanobacterial Culture Collection, PCC, Pasteur Culture Collection, NIES, National Institute for Environmental Studies Microbial Culture Collection, Japan, CCAP Culture Collection of Algae and Protozoa (Windermere, UK).

^*&*^GenBank accession numbers for the microcystin synthetase genes analyzed. For each strain, the upper acc. no. indicates *mcyB *sequence.

* For *Microcystis *strains, the type of McyB A domain, B-type or C-like is indicated by *B *and *C*, respectively.

ND – no data available

### Microcystin isoforms produced by different genera

In total, we identified 22 structural variants (Table 1), mainly differing in the methylation status of D-Asp^3 ^or Dha^7^, but also the amino acid present at position X (Figure 1). Seven different amino acid residues were found at position X, mainly Leu, Arg and homotyrosine (Hty), but also Phe, homoisoleucine (Hil), homophenylalanine (Hph) and Tyr, while only Arg was found at position Z (Table 1).

The *Anabaena *strains mainly produce MC-LR variants, but also several other isoforms, e.g. MC-RR and MC-HtyR (Table 1). Nine *Microcystis *strains produce MC-RR, either as the only isoform or together with MC-LR and/or MC-YR. One strain produces only MC-YR isoforms, while seven strains produce MC-LR isoforms (together with MC-YR for two strains) (Table 1). Two of the *Microcystis *strains examined here have a partial deletion of the *mcy *gene cluster and do not produce microcystin [13]. Within *Planktothrix*, 15 of 18 strains produce MC-RR, either as the only isoform (5 strains), together with MC-LR (8 strains) or together with MC-LR and MC-HtyR (2 strains). The remaining three strains produce mainly MC-HtyR, one of them together with MC-LR (Table 1).

### Adenylation domain-encoding sequences of *mcyB1 *and *mcyC*

Analysis of the McyB1 sequences from *Anabaena *and *Planktothrix *revealed the presence of a single type of A domain with a high degree of sequence similarity to the A domain of McyC. Among *Microcystis*, 7 strains were found to possess a B-type (activating mainly Leu) and 12 strains a C-like (activating mainly Arg – like the McyC) A domain in McyB1 (Table 1, indicated by B and C, respectively). Phylogenetic analyses of cyanobacterial A domains have shown that the McyB1 sequence from *Microcystis *strain PCC 7806 (which is of B-type) does not cluster with McyC A domain sequences as do McyB1 sequences from *Anabaena *and *Planktothrix*, but clusters with other Leu activating A domains [3,19]. Phylogenetic analysis of 115 McyB1 and McyC A domain sequences aligned with the remaining Mcy adenylation domains from *Microcystis*, *Anabaena *and *Planktothrix *(acc. nos. AF183408, AJ536156 and AJ441056, respectively) showed also that McyB1 B-type A domains of *Microcystis *form a clade separate from other McyB1 and McyC sequences (Additional file 1, Figure S1). Therefore, in the comparisons below of McyB1 sequences from all three genera, only the C-like McyB1 sequences of *Microcystis *were included.

The variation measured as percentage divergence and nucleotide diversity (π) within the *mcyB1 *sequences was similar in *Anabaena *(0–6%, π = 0.032) and *Microcystis *(0–6.3%, π = 0.033) and slightly lower (0–3.6%, π = 0.023) in the *Planktothrix *data set. The sequence variation within *mcyC *was low in *Anabaena *(0–2.4%, π = 0.009) and *Planktothrix *(0–1.2%, π = 0.003) and similar to that of *mcyB1 *in the *Microcystis *data set (0–7.1%, π = 0.035) (Table 2). When C-like A domain encoding sequences in *mcyB1 *were compared with the *mcyC *sequence from the same strain, genus-specific differences in the genetic variation were observed: 0.7–7.2% (π = 0.037) in *Anabaena *strains, 8.6–12.4% (π = 0.035) in *Microcystis *strains and 29.8–30.8% (π = 0.158) in *Planktothrix *strains (Table 2). Interestingly, the sequences from the non-producing *Microcystis *strains did not diverged from the rest, suggesting insufficient time for any divergence or that the selective constraints still are the same.

**Table 2:** Genetic information

Genus	No of seq	Length (bp)	π	Sequence variation	No of segregating sites/informative sites	Putative recombination events		
						
						Mosaic structure of informative sites	Detected by programs of RDP2 package	Detected by SplitsTree (Phi test for recomb)
***Anabaena***								
*mcyB*	21	1068	0.032	0–6%	100/79	Y	Y	Y, (*P *< 0.01)
*mcyC*	21	1068	0.009	0–2.4%	31/28	Y	N	Y, (*P *< 0.01)
*mcyBC*	42	1068	0.036	0.7–7.2%*	107/94	Y	Y	Y, (*P *< 0.01)

***Microcystis***								
*mcyB*	12	1059/1062	0.033	0–6.3%	111/57	Y	Y	Y, (*P *< 0.01)
*mcyC*	18	1059/1062	0.035	0–7.1%	131/99	Y	Y	Y, (*P *< 0.01)
*mcyBC*	30	1059/1062	0.073	8.6–12.4%*	222/192	Y	Y	Y, (*P *< 0.01)

***Planktothrix***								
*mcyB*	18	1080	0.023	0–3.6%	61/61	Y	Y	Y, (*P *< 0.01)
*mcyC*	18	1068	0.003	0–1.2%	27/0	N	N	N
*mcyBC*	36	1068/1080	0.158	29.8–30.8%*	354/353	N	N	Y, (*P *< 0.01)^&^

**Comparison of adenylation domains between genera**								
*mcyB1*	51	1062–1080	0.206	27–34%^#^	487/487	N^‡^	N^‡^	N^‡^
*mcyC*	57	1068	0.161	18–29%^§^	411/411	N^‡^	N^‡^	N^‡^

π Nucleotide diversity – the average number of nucleotide differences per site between two sequences

* Sequence variation between *mcyB1 *and *mcyC *sequences

^# ^Sequence variation is 27–30% between *Anabaena *and *Microcystis*, 30–32% between *Anabaena *and *Planktothrix*, 32–34% between *Microcystis *and *Planktothrix*.

^§ ^Sequence variation is 26–29% between *Anabaena *and *Microcystis*, 18–19% between *Anabaena *and *Planktothrix*, 23–26% between *Microcystis *and *Planktothrix*.

^‡ ^Recombination detection between genera

^&^Recombination detected within *mcyB1*

### Variation in evolutionary rates between genera and between McyB1 and McyC

**Figure 2:**
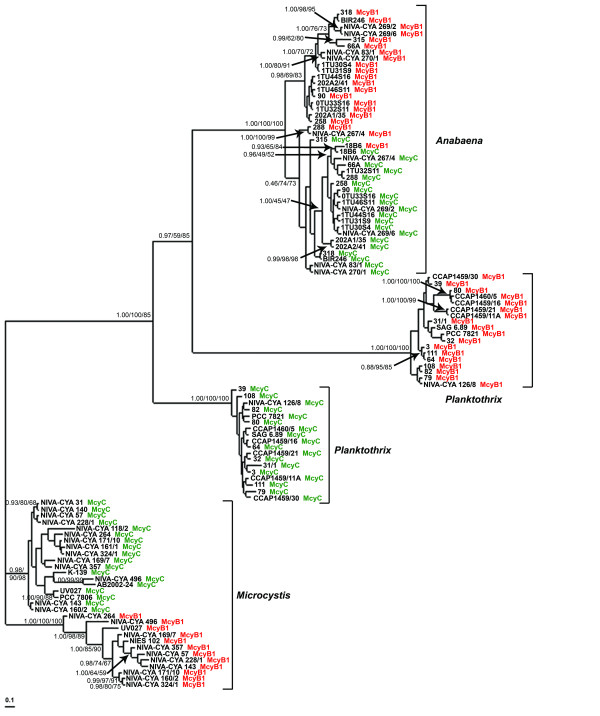
**(A) Phylogenetic analysis of adenylation domains of McyB1 and McyC.** The Bayesian tree is shown with support from maximum likelihood tree (1000 replicates and neighbor-joining tree (1000 replicates). Bayesian posterior probability/ML bootstrap/NJ bootstrap values are shown. Only bootstrap values above 50% are shown. Adenylation domains of McyB1 and McyC from all genera are indicated by red and green, respectively.

Phylogenetic analyses of the amino acid sequence alignment of the 108 McyB1 and McyC A domain sequences yielded a similar tree topology for all methods used (ML, Bayesian and NJ, Figure 2). Five well-supported main clades were observed: McyB1/McyC of *Anabaena*, McyB1 of *Microcystis*, McyC of *Microcystis*, McyB1 of *Planktothrix *and McyC of *Planktothrix*. All clades were genus-specific. The McyB1 and McyC sequences from *Microcystis *formed two separate clades, as did the McyB1 and McyC sequences from *Planktothrix*, but the A domains of *Planktothrix *were separated on longer branches. Interestingly, within the *Anabaena *clade, no distinct, well-supported McyC clade was inferred. Almost all *Anabaena *McyB1 sequences formed a clade with moderate support (PP 0.98, BS-ML 69%, BS-NJ 83%), except for the sequences from strains 288, N-C 267/4 and 18B6. The McyB1 sequence from strain 18B6 clustered with the McyC sequence from the same strain with moderate support (PP 0.93, BS-ML 65%, BS-NJ 84%).

### Mutation rates and recombination within- and between McyB1 and McyC adenylation domains

The mutation rates ranged from 0.0076 to 0.0359, being lowest in *mcyC *of *Anabaena *and *Planktothrix *(Table 3). Moderate recombination levels (0.010 ≤ ρ ≤ 0.027 per base) (Table 3) were detected in all data sets except for *mcyC *from *Planktothrix*. Low recombination levels were estimated for this data set, but all three permutation tests indicated that recombination rate was not statistically significant different from 0. Recombination rate/mutation rate ratios below 1 in the *Microcystis *and *Planktothrix *data sets (Table 3) suggest that point mutations are the main cause of genetic variation in McyB1 and McyC A domains from these genera. In contrast, a recombination rate/mutation rate ratio higher than 1 in the *Anabaena *data sets indicates that recombination has had a major impact on these A domains.

**Figure 3:**
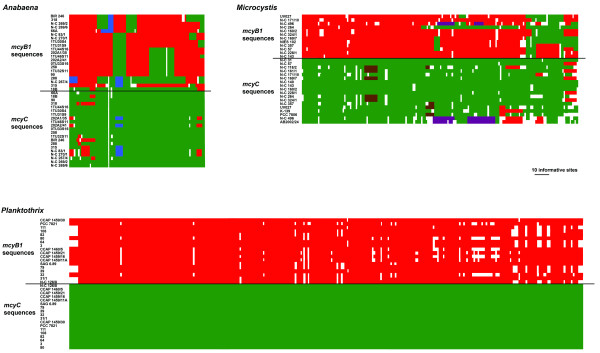
**Informative sites in *Anabaena*, *Microcystis *and *Planktothrix mcyB1C *data sets.** Informative sites are defined as positions with at least two different nucleotides in which each of the variants occurs at least twice. Identical nucleotides have the same colour and the colours thus display phylogenetic affinity.

**Figure 4:**
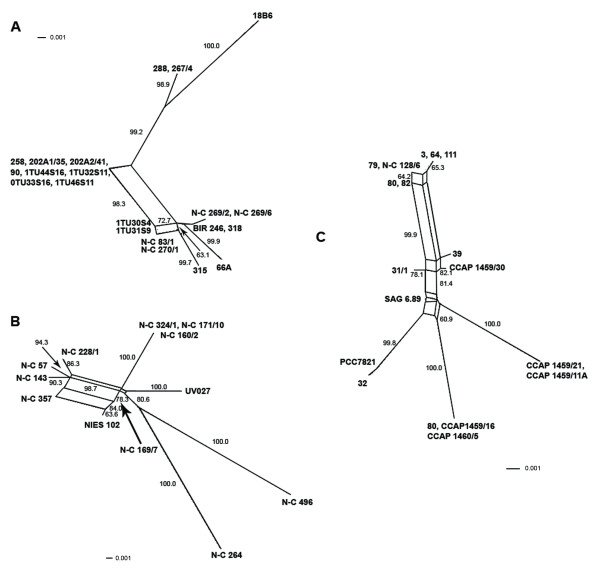
**Splits decomposition analysis of adenylation domain encoding sequences of *mcyB1*.** Shown are *Anabaena *(A), *Microcystis *(B) and *Planktothrix *(C). Bootstrap values over 50% are shown.

**Figure 5:**
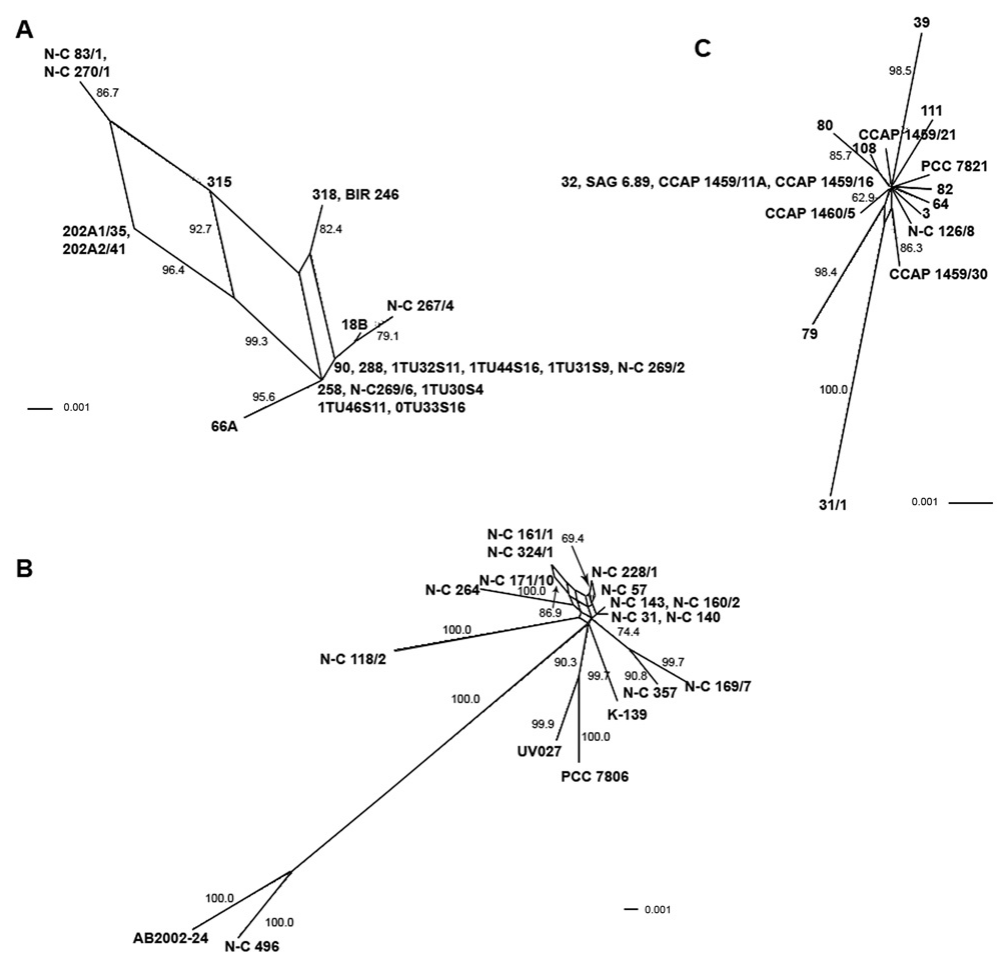
**Splits decomposition analysis of adenylation domain encoding sequences of *mcyC*.** Shown are *Anabaena *(A), *Microcystis *(B) and *Planktothrix *(C). Bootstrap values over 50% are shown.

**Figure 6:**
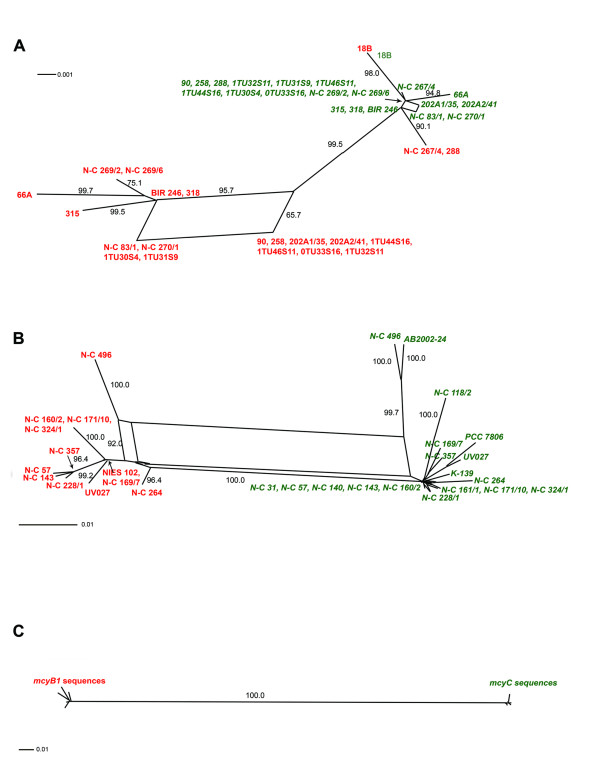
**Splits decomposition analysis of adenylation domain encoding sequences of *mcyB1 *and *mcyC*.** Shown are *Anabaena *(A), *Microcystis *(B) and *Planktothrix *(C). *mcyB1 *and *mcyC *sequences are indicated by red and green, respectively. Bootstrap values above 50% are shown. Within *mcyC *sequences of *Microcystis*, all branches have bootstrap values ranging from 88–100%.

Recombination events were also suggested in all data sets by the mosaic structure of informative sites, with the exception of *mcyC *from *Planktothrix *(Figure 3). The reticulate phylogenies revealed by the split decomposition analysis (Figures 4 and 5) were supported by Phi test (Table 2) in all data sets except *mcyC *from *Planktothrix*. Recombination detection programs (RDP, GENECONV and MaxChi) identified several recombination breakpoints along the entire *mcyB1 *sequences in *Anabaena *and *Planktothrix *strains, while only one single putative recombination event was detected within the *Microcystis mcyB1 *and *mcyC *data sets (Table 4). No recombination events were suggested by recombination detection programs within the *mcyC *alignments of *Anabaena *and *Planktothrix*. The analyses of the combined *mcyB1C *data sets (Figure 6, Table 5) suggested recombination events between *mcyB1 *and *mcyC *in *Anabaena *and *Microcystis*, but not in *Planktothrix*.

**Table 3:** Recombination and mutation rates

Genus	Region analyzed	ρ^a^	Θ_W_^a^	ρ/Θ_W_
*Anabaena*	*mcyB1*	0.0234**	0.0206	1.136
*Anabaena*	*mcyC*	0.0178*	0.0086	2.070
*Microcystis*	*mcyB1*	0.0226**	0.0346	0.653
*Microcystis*	*mcyC*	0.0273**	0.0359	0.760
*Planktothrix*	*mcyB1*	0.0102**	0.0164	0.622
*Planktothrix*	*mcyC*	0.0019^#^	0.0076	0^#^

^a ^recombination rate (ρ) and mutation rate (Θ_W_) per base

* *P *< 0.05 for at least two of three permutation tests implemented in LDhat package

** *P *< 0.001 for at least two of three permutation tests implemented in LDhat package

^# ^All three permutation tests suggested that recombination rate is not significantly different form 0.

**Table 4:** Recombination detected within *mcyB1 *and *mcyC *data sets by RPD, GENECONV and MAXCHI2

Strains involved	RDP fragment, *P *value	GENECONV		MaxChi, fragment, *P *value
			
		(g = 1) fragment, *P *value	(g = 0) fragment, *P *value	
**Putative recombination events detected within *mcyB1***				

***Anabaena***				
**318 **(BIR246, N-C 269/2, N-C 269/6) **66A**	102–594 0.0015	102–594 0.008	--	--
**18B6** **288**	17–457 <0.001	17–457 0.013	--	17–457 <0.001
**66A **(315, BIR246) **1TU30S4**	828–1062 <0.001	828–1062 0.026	828–1062 <0.001	--
**N-C 269/6 **(N-C 269/2, 315) **18B6 **(1TU44S16)	528–742 0.05	528–742 <0.001	528–742 <0.001	528–742 <0.001
**N-C 83/1 **(1TU31S9, 1TU30S4) **1TU46S11**	777–858 <0.001	777–858 <0.001	--	--
**315** **288**	725–997 0.016	725–997 0.0015	725–997 <0.001	725–997 <0.001

***Microcystis***				
**N-C 357 **(N-C 57, N-C 143, N-C 228/1) **NIES 102**	879–925 <0.001	879–925 <0.001	879–925 <0.001	879–925 <0.001

***Planktothrix***				
**CCAP 1459/30** **31/1**	393–606 0.026	--	--	393–606 0.006
**111 **(3, 64) **SAG 6.89**	755–843 0.008	607–884 0.048	607–884 0.02	--
**79** **CCAP 1459/30**	567–843 0.0125	--	520–877 0.013	550–843 <0.001
**79** **31/1**	878–1050 0.016	875–1080 0.05	875–1080 0.016	--
**N-C 126/8** **80**	--	520–877 0.05	520–877 0.009	550–843 <0.001

**Putative recombination events detected within *mcyC***				

***Anabaena***	--	--	--	--

***Microcystis***				
**N-C 161/1 **(N-C 171/10, N-C 324/1, N-C 264) **N-C 228/1**	1–203 0.0016	1–246 0.019	--	--

***Planktothrix***	--	--	--	--

Events detected by two or more methods are listed.

**Table 5:** Recombination detected between *mcyB1 *and *mcyC *by RPD, GENECONV and MAXCHI2

Strains involved	RDP fragment, *P *value	GENECONV		MaxChi, fragment, *P *value
			
		(g = 1) fragment, *P *value	(g = 0) fragment, *P *value	
***Anabaena***				
**McyB1 **288 **McyC **66A	--	132–426, 0.001	132–429, <0.001	132–426, <0.001
**McyB1 **1TU44S16 **McyC **66A (18B6, 90, 318, 258, 202A1/35, 1TU44S16, 1TU32S11, 288, 202A2/41, 0TU33S16, 315, BIR246, N-C 267/4, N-C 269/2, N-C 269/6)	742–864, <0.001	742–864, <0.001	--	742–864, <0.001
**McyB1 **st 288 **McyC **202A1/35 (202A2/41)	--	311–432 0.0014	311–432, 0.0011	311–432, <0.001
**McyB1 **1TU46S11 **McyC **N-C 83/1 (N-C 270/1)	742–864 <0.001	742–864 <0.001	--	--
**McyB1 **258 **McyC **N-C 269/6	--	103–456, 0.001	103–456, <0.001	80–599, <0.001

***Microcystis***				
**McyB1 **N-C 264 **McyC **N-C 31 (N-C 57, N-C 140, N-C 143, N-C 160/2)	1–279, 879–1062, <0.001	1–279, 879–1062, <0.001	36–210, <0.001	--
**McyB1 **N-C 264 **McyC **N-C 31 (N-C 57, N-C 357)	707–918, <0.001	707–918, <0.001	795–1056 0.0013	707–918, <0.001
**McyB1 **N-C 264 **McyC **N-C 161/1	270–795, <0.001	262–765, <0.001	--	236–1056, <0.001
**McyB1 **N-C 264 **McyC **N-C 169/7 (N-C 171/10 N-C 264, N-C 357, N-C 496)	1–279, 879–1062, <0.001	3–270, 879–1062 <0.001	3–270, <0.001	3–270, <0.001
**McyB1 **N-C 264 **McyC **N-C 171/10 (N-C 324/1, UV027, N-C 140, N-C 143, N-C 160/2)	466–782, 0.00105	466–782, 0.009	466–782, <0.001	--
**McyB1 **N-C 264 **McyC **N-C 228/1	--	36–210, <0.001	--	36–210, <0.001
**McyB1 **N-C 264 **McyC **N-C 228/1	319–766, <0.001	319–766, <0.001	--	319–766, <0.001
**McyB1 **N-C 169/7 **McyC **N-C 496	--	238–469, <0.001	--	238–469, <0.001
**McyB1 **N-C 264 **McyC **N-C 496, UV027, K-139, PCC7806	--	3–270, <0.001	3–270, <0.001	3–270, <0.001
**McyB1 **N-C 57 **McyC **N-C 143, K-139	444–769, <0.001	444–769, <0.001	444–769, <0.001	--
**McyB1 **N-C 169/7 **McyC **K-139	707–915, <0.001	707–915, <0.001	--	--
**McyB1 **N-C 357 **McyC **N-C 264	--	368–779, <0.001	--	368–779 <0.001
**McyB1 **N-C 357 **McyC **N-C 171/10	--	879–925, <0.001	--	879–925, <0.001
**McyB1 **N-C 228/1 (NIES102) **McyC **N-C 160/2 (N-C 143)	368–779, <0.001	368–779, <0.001	368–792, <0.001	--

***Planktothrix***	--	--	--	--

Events detected by two or more methods are listed.

### Substrate specificity of MycB1 and McyC adenylation domains

McyB1 and McyC A domain sequences were aligned with the Phe-activating A domain of GrsA [20] to identify the binding-pocket residues. The binding pocket signatures of McyC A domains (activating mainly Arg) were more or less identical within the genus, while only five residues are identical in binding pocket signatures from all three genera (Table 6). Binding pocket signatures are more diverse in McyB1 A domains, reflecting the diversity of amino acid residues incorporated in position X (Table 6). Some McyB1 modules with identical binding pocket signatures incorporate a somewhat different set of amino acid residues (e.g. *Microcystis *strains N-C 357 and N-C 496, Table 6), indicating that other residues in the A domain or other domains, such as the condensation domain influence substrate specificity. A role of the condensation domain in substrate selection has been suggested by several studies (for a review, see [18]).

**Table 6:** Binding pocket signatures identified in A domain sequences.

Strain	Adenylation domain of McyB1										Adenylation domain of McyC									
	
	Binding pocket residues									Substrate*	Binding pocket residues									Substrate*
				
	235	236	239	278	299	301	322	330	331		235	236	239	278	299	301	322	330	331	
***Anabaena***																				
N-C 83/1	D	V	W	**F**	F	G	L	V	**D**	Leu, Arg	D	V	W	**S**	F	G	L	V	D	Arg
N-C 267/4	D	V	W	**C**	F	G	L	V	**Y**	Hty, Leu, Phe, Hil, Hph	D	V	W	**S**	F	G	L	V	D	Arg
N-C 269/2	D	V	W	**C**	F	G	L	V	**Y**	Hty, Leu, Phe, Hil, Hph	D	V	W	**C**	F	G	L	V	D	Arg
N-C 269/6	D	V	W	**C**	F	G	L	V	**Y**	Hty, Leu, Phe, Hil, Hph	D	V	W	**C**	F	G	L	V	D	Arg
N-C 270/1	D	V	W	**F**	F	G	L	V	**D**	Leu, Arg	D	V	W	**S**	F	G	L	V	D	Arg
90	D	V	W	**F**	F	G	L	V	**D**	Leu, Arg	D	V	W	**C**	F	G	L	V	D	Arg
1TU44S16	D	V	W	**F**	F	G	L	V	**D**	Leu	D	V	W	**C**	F	G	L	V	D	Arg
1TU30S4	D	V	W	**F**	F	G	L	V	**D**	Leu	D	V	W	**C**	F	G	L	V	D	Arg
1TU31S9	D	V	W	**F**	F	G	L	V	**D**	Leu	D	V	W	**C**	F	G	L	V	D	Arg
202A1/35	D	V	W	**F**	F	G	L	V	**D**	Leu	D	V	W	**S**	F	G	L	V	D	Arg
1TU46S11	D	V	W	**F**	F	G	L	V	**D**	Leu	D	V	W	**C**	F	G	L	V	D	Arg
202A/41	D	V	W	**F**	F	G	L	V	**D**	Leu	D	V	W	**S**	F	G	L	V	D	Arg
0TU33S16	D	V	W	**F**	F	G	L	V	**D**	Leu	D	V	W	**C**	F	G	L	V	D	Arg
258	D	V	W	**F**	F	G	L	V	**D**	Leu	D	V	W	**C**	F	G	L	V	D	Arg
1TU32S11	D	V	W	**F**	F	G	L	V	**D**	Leu	D	V	W	**S**	F	G	L	V	D	Arg
BIR 246	D	V	W	**C**	F	G	L	V	**Y**	Hty, Leu, Hil, Phe, Hph	D	V	W	**C**	F	G	L	V	D	Arg
288	D	V	W	**C**	F	G	L	V	**Y**	Hty, Leu, Phe, Hph	D	V	W	**S**	F	G	L	V	D	Arg
315	D	V	W	**S**	F	G	L	V	**Y**	Leu, Hty	D	V	W	**S**	F	G	L	V	D	Arg
318	D	V	W	**C**	F	G	L	V	**Y**	Hty, Leu	D	V	W	**C**	F	G	L	V	D	Arg
66A	D	V	W	**S**	F	G	L	V	**Y**	Hph, Hty, Leu	D	V	W	**S**	F	G	L	V	D	Arg
18B6	D	V	W	**S**	F	G	L	V	**D**	Arg	D	V	W	**S**	F	G	L	V	D	Arg

***Microcystis***																				
N-C 31											D	V	W	T	I	G	A	V	D	Arg
N-C 57	D	**G**	W	T	I	G	A	V	**E**	Arg	D	V	W	T	I	G	A	V	D	Arg
N-C 118/2											D	V	W	T	I	G	A	V	D	Arg
N-C 140											D	V	W	T	I	G	A	V	D	Arg
N-C 143	D	**G**	W	T	I	G	A	V	**E**	None	D	V	W	T	I	G	A	V	D	Arg
N-C 160/2	D	**G**	W	T	I	G	A	V	**E**	None	D	V	W	T	I	G	A	V	D	Arg
N-C 161/1											D	V	W	T	I	G	A	V	D	Arg
N-C 169/7	D	**G**	W	T	I	G	A	V	**E**	Arg, Leu	D	V	W	T	I	G	A	V	D	Arg
N-C 171/10	D	**G**	W	T	I	G	A	V	**E**	Leu, Arg, Tyr	D	V	W	T	I	G	A	V	D	Arg
N-C 228/1	D	**G**	W	T	I	G	A	V	**E**	Arg, Leu	D	V	W	T	I	G	A	V	D	Arg
N-C 264	D	**V**	W	T	I	G	A	V	**D**	Arg	D	V	W	T	I	G	A	V	D	Arg
N-C 324/1	D	**G**	W	T	I	G	A	V	**E**	Arg, Leu	D	V	W	T	I	G	A	V	D	Arg
N-C 357	D	**G**	W	T	I	G	A	V	**E**	Arg, Leu, Tyr	D	V	W	T	I	G	A	V	D	Arg
N-C 496	D	**G**	W	T	I	G	A	V	**E**	Tyr	D	V	W	T	I	G	A	V	D	Arg
AB2002-24											D	V	W	T	I	G	A	V	D	Arg
UV027	D	**V**	W	T	I	G	A	V	**E**	Arg	D	V	W	T	I	G	A	V	D	Arg
PCC7806											D	V	W	T	I	G	A	V	D	Arg
K-139											D	V	W	T	I	G	A	V	D	Arg
NIES102	D	**G**	W	T	I	G	A	V	**E**	Leu, Arg, Tyr	D	V	W	T	I	G	A	V	D	Arg

***Planktothrix***																				
3	D	A	**L**	**F**	F	G	**V**	V	**D**	Arg	D	P	W	G	F	G	L	V	D	Arg
64	D	A	**L**	**F**	F	G	**V**	V	**D**	Arg	D	P	W	G	F	G	L	V	D	Arg
111	D	A	**L**	**F**	F	G	**V**	V	**D**	Arg	D	P	W	G	F	G	L	V	D	Arg
31/1	D	A	**L**	**F**	F	G	**L**	V	**D**	Arg, Hty, Leu	D	P	W	G	F	G	L	V	D	Arg
32	D	A	**L**	**F**	F	G	**L**	V	**D**	Arg, Leu	D	P	W	G	F	G	L	V	D	Arg
39	D	A	**L**	**F**	F	G	**L**	V	**D**	Arg, Leu	D	P	W	G	F	G	L	V	D	Arg
79	D	A	**L**	**F**	F	G	**L**	V	**D**	Arg, Leu	D	P	W	G	F	G	L	V	D	Arg
SAG 6.89	D	A	**L**	**F**	F	G	**L**	V	**D**	Arg, Leu	D	P	W	G	F	G	L	V	D	Arg
N-C 126/8	D	A	**L**	**F**	F	G	**L**	V	**D**	Arg, Leu	D	P	W	G	F	G	L	V	D	Arg
80	D	A	**L**	**L**	F	G	**F**	V	**A**	Hty	D	P	W	G	F	G	L	V	N	Arg
82	D	A	**L**	**F**	F	G	**L**	V	**D**	Arg, Hty, Leu	D	P	W	G	F	G	L	V	D	Arg
108	D	A	**L**	**F**	F	G	**L**	V	**D**	Arg, Leu	D	P	W	G	F	G	L	V	D	Arg
PCC7821	D	A	**L**	**F**	F	G	**L**	V	**D**	Arg, Leu	D	P	W	G	F	G	L	V	D	Arg
CCAP1459/30	D	A	**L**	**F**	F	G	**L**	V	**D**	Arg, Leu	D	P	W	G	F	G	L	V	D	Arg
CCAP1459/11A	D	A	**W**	**F**	F	G	**L**	V	**D**	Arg	D	P	W	G	F	G	L	V	D	Arg
CCAP1459/21	D	A	**W**	**F**	F	G	**L**	V	**D**	Arg	D	P	W	G	F	G	L	V	D	Arg
CCAP1460/5	D	A	**L**	**L**	F	G	**F**	V	**A**	Hty, Leu	D	P	W	G	F	G	L	V	D	Arg
CCAP1459/16	D	A	**L**	**L**	F	G	**F**	V	**A**	Hty, Leu	D	P	W	G	F	G	L	V	D	Arg

*According to isoforms produced

### Adenylation domains and selective forces

**Figure 7:**
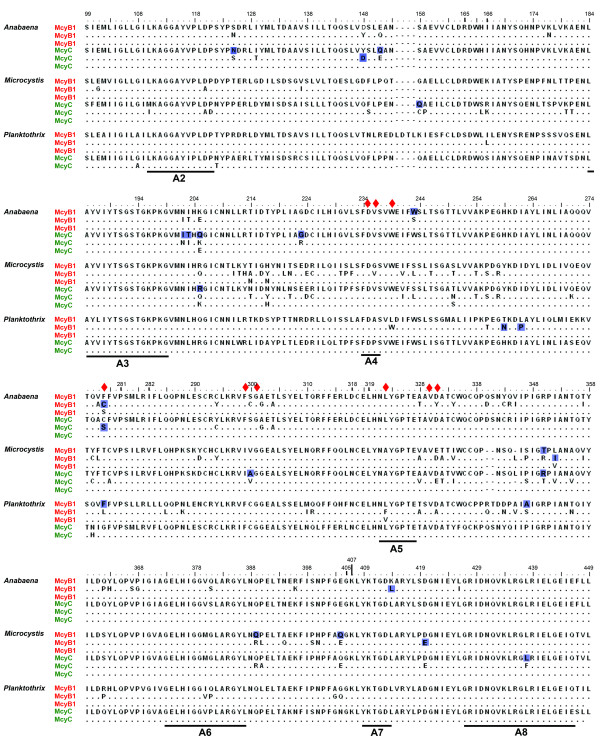
**Alignment of adenylation domain sequences of McyB1 and McyC in *Anabaena*, *Microcystis *and *Planktothrix *strains.** Identical amino acid residues within genus sequences are indicated by •. Positions of the conserved motifs [2] are shown and binding pocket residues [32] are indicated by red diamonds. Amino acid residues undergoing positive selection are shown in dark blue boxes. Numbering of amino acid residues according to GrsA (swissprot: P0C061).

An excess of synonymous over non-synonymous substitutions (ω < 1) (Table 7) was observed in all data sets, indicating that the A domains of McyB1 and McyC overall are under purifying selection in all three genera. Small fractions (0.3–10.4%) of codons under positive selection were detected in all data sets except for McyC from *Planktothrix *(Table 7). The number of potential sites under positive selection with statistical support (P > 90%) ranged from 3 to 8 (Table 7) and their positions in the A domain alignment are shown in Figure 7. Interestingly, in both *Anabaena *data sets as well as in the *Planktothrix *McyB1 data set, the binding pocket residue 278 (Figure 7, Table 7) appears to be under positive selection. In the *Microcystis *McyC data set, this is also the case for the amino acid residue between binding pocket residues 299 and 301 (Figure 7). Among residues not present in binding pocket signatures, site 205 in the McyC alignments in both *Microcystis *and *Anabaena *and site 350 in both *Microcystis *data sets (Figure 7) were suggested to be under positive selection.

**Table 7:** Likelihood ratio tests of positive selection

Genus	Region analyzed	Model	lnL	Estimates of parameters	ω^§^	Positively selected sites^#^	LRT
***Anabaena***	***mcyB1***	M7 (beta)	-2249.981	p = 0.005, q = 0.01858		Not allowed	40.966***
		M8 (beta and ω)	-2229.498	p_0 _= 0.997, p = 0.005, q = 0.021 p_1 _= 0.003, ω = 94.065	0.492	**243W, **ω = 3.717* 278C, ω = 3.935** 414L, ω = 4.053***	
***Anabaena***	***mcyC***	M7 (beta)	-1695.094	p = 0.005, q = 0.0471		Not allowed	20.476***
		M8 (beta and ω)	-1684.856	p_0 _= 0.945, p = 0.005, q = 2.205 p_1 _= 0.055, ω = 6.562	0.362	**125N, **ω = 6.994* 148D, ω = 8.098*** 151Q, ω = 7.518** 202I, ω = 6.992* 203T, ω = 7.240** 205Q, ω = 7.240*** 223G, ω = 7.177** 278S, ω = 7.176**	
***Microcystis***	***mcyB1***	M7 (beta)	-2321.128	p = 0.005, q = 0.016		Not allowed	6.729**
		M8 (beta and ω)	-2318.527	p_0 _= 0.942, p = 0.110, q = 0.773 p_1 _= 0.058, ω = 2.584	0.356	**350T, **ω = 2.879*** 352I, ω = 2.792*** 389Q, ω = 2.727* 404Q, ω = 2.744* 420E, ω = 2.684*	
***Microcystis***	***mcyC***	M7 (beta)	-2674.333	p = 0.012, q = 0.0416		Not allowed	16.516***
		M8 (beta and ω)	-2666.075	p_0 _= 0.976, p = 0.015, q = 0.0811 p_1 _= 0.024, ω = 4.939	0.280	**158Q, **ω = 3.471*** 205R, ω = 3.472*** 300A, ω = 3.279* 349R, ω = 3.481*** 438L, ω = 3.473***	
***Planktothrix***	***mcyB1***	M7 (beta)	-2030.973	p = 0.005, q = 0.021		Not allowed	7.93***
		M8 (beta and ω)	-2027.008	p_0 _= 0.896, p = 0.005, q = 1.883 p_1 _= 0.104, ω = 2.134	0.231	**259N, **ω = 3.074*** 262P, ω = 3.187*** 278F, ω = 3.005*** 347A, ω = 3.000*	
***Planktothrix***	***mcyC***	M7 (beta)	-1595.526	p = 50.84, q = 99.000		Not allowed	0.002
		M8 (beta and ω)	-1595.525	p_0 _= 1.000, p = 50.442, q = 99.00 p_1 _= 0.000, ω = 0.539	0.196	None	

^§ ^calculated using estimates of parameters of best fitting model

^# ^numbering of amino acid residues according to GrsA (swissprot: P0C061)

*90% confidence interval level

** 95% confidence interval level

*** 99% confidence interval level

Branch-site models were used to detect possible positive selection acting on the McyB1 sequences from *Anabaena *and *Planktothrix *strains that incorporate Hty in position X. (Table 1, Figure 2). There were no statistically significant differences between the log-likelihood values of the alternative models and the null models (data not shown), indicating no evidence for positive selection in domains incorporating Hty.

## Discussion

This study is so far the most extensive comparative analysis of microcystin synthetase adenylation domains for the modules McyB1 and McyC. The phylogenetic trees of the 108 adenylation domain sequences showed clustering according to module and genus (Figure 2). Our data set revealed no signs of recombination between genera, in agreement with previous studies on *mcy *genes [3,21] and similar studies from other NRPS gene clusters [19,22]. This also is in line with other studies that show that the rate of successful homologous recombination rapidly is reduced with increased genetic distance [23-25].

### The evolutionary history of the McyB1 A domain

It is not clear at present which of the two types of A domains in McyB1, was present in the ancestral microcystin synthetase. A B-type ancestral A domain implies that after segregation of the genera, some *Microcystis *strains and all *Anabaena *and *Planktothrix *acquired a C-like type of A domain in McyB1, most likely through intragenomic recombination between *mcyB1 *and *mcyC*, as suggested for *Hapalosiphon hibernicus *and *Anabaena *strain 18B6 [3]. If the ancestral McyB1 A domain was C-like, some *Microcystis *strains must have obtained a novel McyB1 A domain, presumably through recombination with a different NRPS gene cluster. Recently, the presence of a Leu-activating, B-type McyB1 A domain was reported in two *Nostoc *strains [3] and this may in contrast strengthen the hypothesis that the ancestral A domain in McyB1 was B-type. However, the B-type A domain sequences from *Microcystis *and *Nostoc *seem to be separated by rather long phylogenetic distances, suggesting that these A domains were introduced in the McyB1 module by two independent recombination events [3]. Clearly, further studies are needed to clarify the evolutionary history of the McyB1 A domain.

### Genomic processes reshaping the adenylation domains of McyB1 and McyC

Our results suggest that recombinations as well as point mutations contribute to variation in the A domains of modules McyB1 and McyC. Within *Anabaena *and *Microcystis*, frequent recombination was suggested both within and between *mcyB1 *and *mcyC *sequences (Figures 3, 4 and 5, Tables 4 and 5). The low sequence variation (0–1.2%) within *Planktothrix mcyC *sequences makes it difficult to detect recombination, since for the majority of methods, a minimum sequence variation of 5% is necessary to obtain substantial power [26]. The large sequence divergence between *Planktothrix mcyB1 *and *mcyC *sequences might prevent homology-driven recombination, which requires a relatively high level of sequence similarity between the donor and recipient DNA. In *Planktothrix*, the longest identical DNA segment shared by *mcyB1 *and *mcyC *(18 bp) may be too short for initiation of RecA-mediated recombination [27-29]. Within *Anabaena *and *Microcystis*, the high sequence similarity between these gene segments appears to be maintained by frequent recombination events. Recombination between different domains, such as *mcyB1 *and *mcyC*, has in some cases lead to replacement of a nearly entire A domain (in *Anabaena *18B6) and in others to replacement of a functionally important part of the domain in McyB1 (in *Microcystis *N-C 264) (Figure 3). In both cases this has resulted in a change of functionality (i.e. amino acid activated) and subsequent production of microcystin-RR.

Our results, together with previous reports [3,11,15], indicate that various types of recombination lead to a continual restyling (remodelling) of the adenylation domains of microcystin synthetase. Recombination within a single domain appears to be frequent and may have little impact on the type of amino acid activated. Recombination between *mcyB1 *and *mcyC *appears to be frequent in some genera and may result in changes in the microcystin profile of the recombinant strains. Successful recombination between A domain regions from different NRPS gene clusters [14,15] were found to be infrequent in the strains investigated here.

### Positive selection in the adenylation domains of McyB1 and McyC

Overall, the adenylation domains of McyB1 and McyC seem to be under purifying selection, as shown previously for other segments of the *mcy *gene cluster [11,12,21], indicating that mutations that affect the amino acid sequence of these domains generally are deleterious. However, the ω-values (0.2–0.49) observed in this study are relatively high compared to ω-values reported for several cyanobacterial house keeping genes (mainly below 0.1) [30], implying a relaxation of selective constraints.

Amino acid residues in the A domains of McyB1 and McyC that seem to be under positive selective pressure are located throughout the entire analyzed sequence (Figure 7). Among the positively selected amino acids, residues included in binding pocket signatures are particularly interesting, since they may influence the active site selectivity [31,32]. The amino acid change Phe→Cys in binding pocket position 278 (Table 6) in the McyB1 sequences of *Anabaena *is an example of this. According to the peptide profiles of the *Anabaena *strains (Table 1), this change is associated with the incorporation of Hty/Leu, rather than only Leu (or Leu/Arg). Also, the Leu→Phe exchange at the binding pocket position 278 in the McyB1 sequences of *Planktothrix *(Table 6) is associated with a change in incorporation from Hty to Arg. One could hypothesize that positive selection of these and other residues in the synthetases reflect selection of a particular peptide profile produced by the corresponding strains. Such a causative relationship between these specific genetic changes and phenotypic effects remains to be demonstrated.

Interestingly, a binding pocket residue under positive selection is also present in the McyC sequences of *Anabaena *(Figure 7). Since all McyC modules studied here mainly incorporate Arg, the selection seemingly does not concern gross substrate specificity. Other properties, like NRPS catalytic efficiency or the ability to produce minor variants, might be the properties selected for. Also in McyB1 sequences from all genera there are several positively selected amino acid residues not associated with substrate selectivity, indicating that some other property is selected for in these A domains. This could for instance again be changes in the catalytic efficiency or in the interactions between neighboring domains and modules.

Sequence comparisons show that the A domain of McyC is more conserved than the McyB1 A domain – also reflected by the lack of amino acid variation in position Z of the produced peptides. Within *Planktothrix*, a lower recombination rate and stronger purifying selection compared to *Anabaena *and *Microcystis *indicate stronger functional constraints.

## Conclusion

Our results revealed no clear indications of recombination across the genera, while frequent recombination events both within and between *mcyB *and *mcyC *sequences were detected between strains from same genus, except for *mcyC *from *Planktothrix*. We demonstrate remodelling of *mcyB *and *mcyC *genes including evidence for positive selection acting at some sites, indicating that the microcystin variant profile of a given strain is likely to influence the ability of the strain to interact with its environment.

## Methods

### Bacterial strains

Cyanobacterial strains were grown at the University of Helsinki and Norwegian Institute of Water Research (NIVA) under continuous white light at a photon irradiance of 7 μmol m^-2 ^s^-1 ^in Z8 medium [33].

### Mass spectrometry

Microcystins were extracted from lyophilized biomass collected on glass fiber filters with 50% MeOH as extraction agent. A detailed description of the method can be found in Rohrlack *et al*. [34].

For the identification of microcystins liquid chromatography with mass spectrometric detection (LC-MS/MS) was used. The instrumental setup included a Waters Acquity UPLC System equipped with a Waters Atlantis C18 column (2.1 × 150 mm, 5 μm particle size) and directly coupled to a Waters Quattro Premier XE tandem quadrupole MS/MS detector. The UPLC system was set to deliver a linear gradient from 20% to 60% acetonitrile in water, both containing 0.1% acetic acid, within 8 minutes at a flow rate of 0.25 mL min^-1^. The column and auto sampler temperatures were 20 and 4°C, respectively. At all times, the MS/MS detector was run in positive electrospray mode (ESI+). Other general settings included a source temperature of 120°C, a desolvation temperature of 350°C, a drying gas flow rate of 800 L hour^-1^, a gas flow at the cone of 50 L hour^-1^, and standard voltages and energies suggested by the manufacturer for the ESI+ mode.

To screen extracts for microcystins, the detector was run in total scanning mode for the mass range from 500 to 1100 Da over the entire UPLC gradient. At this stage, the cone voltage was 60 V and the time for one scan 2 seconds. Afterwards, all mass signals, that represented compounds with a molecular mass within the range of 500–1100 Da, were analyzed in fragmentation experiments. To this end, the detector was run in daughter ion scanning mode and the cone voltage and collision cell settings were optimized to obtain as many fragments of the respective compound as possible. In all cases, argon served as collision gas. Microcystins were identified by their typical fragmentation patterns including a number of immonium ions of amino acids, the characteristic Adda side chain fragment (135 Da), and a number of ring fragments. Identification was further supported by comparing fragmentation patterns with those of Microcystin LR, RR and YR standards that have been purchased from Sigma-Aldrich and by using the fragmentation simulation software HighChemMass Frontier (version 3). The precise positions of demethylations in microcystin molecules were not determined.

### DNA extraction, PCR amplification and sequencing

For microcystin-producing *Anabaena *strains supplied by the University of Helsinki strain collection, DNA was extracted from dried cell matter with Qiagen DNeasy Plant Mini Kit (QIAGEN GmbH, Hilden, Germany). Strains from NIVA were lysed according to Chromczynski and Rymaszewski [35] and PCR performed directly on the lysate.

PCR was performed with DynaZyme II DNA polymerase (Finnzymes, Espoo, Finland) and BD Advantage™ 2 polymerase (BD Biosciences, Palo Alto, CA, USA). Primers used for amplification of adenylation domains from the *mcyABC *operon are listed in Table 8 and their relative positions in the *mcyABC *operon are shown in Figure 1. Genus-specific primers for *Microcystis *and *Anabaena *were designed based on the publicly available *mcy *gene sequences of *Microcystis aeruginosa *PCC 7806 (AF183408) and UV027 (AF458094) and *Anabaena *strain 90 (AJ536156). Primers used for amplifying the *mcyB *segment from *Microcystis *strains were placed as far as possible apart from the region involved in the recombination event between the A domain-encoding segments of *mcyB *and *mcyC *[13,15]. The *mcyB *regions flanking the recombination site are highly similar in all *Microcystis *strains (Additional file 1, Figure S1). The PCR products were purified using E.Z.N.A Gel Extraction Kit (Omega Biotek) and Montage™ PCR Centrifugal Filter Devices (Millipore, Billerica, MA, USA). The purified PCR products were sequenced with both external and internal primers (Table 8). Sequencing was conducted under BigDye™ terminator cycling conditions, and sequencing reactions were purified using ethanol precipitation and separated on an Applied Biosystems 3730xl DNA Analyzer. Chromatograms were examined with the program CHROMAS 2.2 (Technelysium Pty Ltd.), while editing and contig assembly were performed with BIOEDIT sequence alignment editor. All sequences have been submitted to GenBank under accession numbers EU009866–EU009922 (Table 1). Several sequences (for *Microcystis *strains PCC 7806, K-139, UV027, NIES102 and *Anabaena *strain 90) were retrived from GenBank together with A domain sequences from *Planktothrix *spp. generated by Kurmayer and co-workers [11,14] (Table 1).

**Table 8:** Primers used.

Primer	Sequence	Annealing temp (C°)
*Microcystis*-mcyB-F (PCR and sequencing)	5'-CCCAAGAGCAACATCAGTTATTAGT-3'	58
*Microcystis*-mcyB-R (PCR and sequencing)	5'-TTCCTGTCTATCTTGCCATTGTTA-3'	57
*Microcystis*-mcyB-F2 (Sequencing)	5'-AACGACTCCTGAGAATTTAGCCTAT-3'	60
*Microcystis*-mcyB-R2 (Sequencing)	5'-GTCAATTCAGGTTGGTTGAGGT-3'	60
*Microcystis*-mcyC-F (PCR and sequencing)*	5'-CAAGAAAAAGGCGTAACTTCAGA-3'	55
*Microcystis*-mcyC-R (PCR and sequencing)*	5'-AAGGTATCTTCCCGCATAATC-3'	55
*Anabaena*-mcyB-F (PCR and sequencing)	5'-TGATTTGAAAAGAAAGACCCAAT-3'	56
*Anabaena*-mcyB-R (PCR and sequencing)	5'-ATACCCAAACAAGAGTTGCTCAT-3'	59
*Anabaena*-mcyB-F2 (Sequencing)	5'-ACTTATCCGCTTATCGCAGGT-3'	56
*Anabaena*-mcyB-R2 (Sequencing)	5'-CCCAATATGTAATTCTCCAGCA-3'	56
*Anabaena*-mcyC-F (PCR and sequencing)	5'-CTCAATTCTGCTACTGTTGGTTTT-3'	57
*Anabaena*-mcyC-R (PCR and sequencing)	5'-CTTACCCACTAAAACCTCGAACT-3'	54
*Anabaena*-mcyC-F2 (Sequencing)	5'-AGGTAAGCCAAAGGGAGTGAT-3'	57
*Anabaena*-mcyC-R2 (Sequencing)	5'-CACCTCCAATATGTAATTCTCCA-3'	57

Primers *Microcystis-mcyC*-F and *Microcystis-mcyC*-R were used in [13]

### Sequence alignments and phylogenetic analyses

Amino acid sequences of A domains from all genera were aligned using ClustalW [36]. The best evolution model based on the sequence alignment was determined using ProtTest [37]. The sequences were used to infer the phylogeny in a Bayesian framework applying the program MrBayes v3.1 [38]. Analysis with the following parameters was performed: JTT model, gamma distribution, running 2 million generations and sampling trees every 100 generation, burn-in 3000 trees. The maximum likelihood (ML) tree was estimated using PhyML [39] under the JTT model, gamma distribution and with parameter values indicated by ProtTest. The Neighbor joining (NJ) tree was obtained under the JTT model and gamma distribution using MEGA version 3 [40]. Bootstrap confidence limits were obtained by 1000 replicates in both ML and NJ analysis. DnaSP version 3.51 [41] was used to estimate mutation rates, based on the number of segregating sites, using the Watterson's estimator of *Θ *[42] and the average nucleotide diversity (π) [43].

### Recombination analyses and nucleotide substitution statistics

Recombination was investigated by split decomposition analysis using SplitsTree version 4.8 [44] with default settings (uncorrected P method) and 1000 bootstrap replicates together with Phi test for recombination [45]. In addition, the following statistical tests for detecting recombination were used: GENECONV [46], RDP, and MaxChi [47] analyses in the RDP version 2 *b*08 program package [48]. For detecting recent and older recombination events using GENECONV G-scale values 0 and 1 were used, respectively. Recombination was also detected by visual analysis of informative sites (variable sites where each variant occurs in at least two sequences) as described by Rudi *et al*. [24].

The recombination rate, ρ = 2 *Nr *(*N *is the effective population size and *r *is the recombination rate per nucleotide site per generation) was estimated for each data set using the composite likelihood method proposed by Hudson [49] and extended to allow for finite-site mutation models [50]. The method is based on combining the coalescent likelihoods of all pairwise comparisons of segregating sites. The hypothesis of no recombination was tested using the likelihood permutation test (LPT) as in McVean *et al*. [50] and the permutation tests which detect a decrease in *r*^2 ^and |*D*'|, measures of linkage disequilibrium, with an increase in the physical distance. Both the composite likelihood analysis and the three permutation tests were carried out using the LDhat package [50].

We used CODEML from the PAML v3.15 package [51] to test for the presence of codon sites affected by positive selection and to identify those sites under selection. A likelihood ratio test (LRT) for positive selection [52,53] compares two codon substitution models, one of which accounts for positive selection and the other which does not. The gene is inferred to be under positive selection if (1) ML estimates suggests that there are codon(s) under positive selection (with ω = d_n_/d_s _> 1) and (2) the LRT is significant. Simulations by Anisimova *et al*. [54] showed that high levels of recombination seem to affect dramatically the accuracy of the LRT test and that recombination often mistakenly is seen as evidence of positive selection. LRTs of M0–M3 and M1–M2 are heavily affected, while LRT of M7–M8 is much less (positive selection was falsely detected in only 20% of replicates). Therefore, models M7 (beta) and M8 (beta and ω) were considered in present study. Under the model M7 (beta), the ω ratio various according to the beta distribution and does not allow the positive selected sites (< ω < 1), and thus serves as the null model by comparing with model M8 (beta and ω). Model M8 adds an additional site class to the beta model to account for sites under positive selection (ω > 1). A Bayesian approach implemented in CODEML and shown to be robust to recombination effects [54] was used to identify residues under positive selection. The average ω for A domain sequences was calculated using the parameters of the best fitting model.

Branch-site models [55] were employed to test for positive selection acting on specific branches in the phylogenetic tree. Branches of the tree were divided *a priori *into foreground and background lineages, and a LRT was constructed by comparing a model that allows positive selection on the foreground lineages (alternative model) with a model that does not allow such positive selection (the null model).

## Abbreviations

BS: bootstrap; LC-MS/MS: liquid chromatography with mass spectrometric detection; MC-LR: leucine and arginine in the positions of X and Z of microcystin; MC-RR: arginine in the positions of X and Z of microcystin; MC-HtyR: homotyrosine and arginine in the positions of X and Z of microcystin; MC-YR: tyrosine and arginine in the positions of X and Z of microcystin; ML: maximum likelihood; NJ: neighbor joining; PP: Posterior Probability.

## Authors' contributions

ATK designed the study, contributed to molecular studies, performed the phylogenetic, recombination, mutation and selection analysis and drafted the manuscript. DPF: Contributed to molecular studies and helped draft the manuscript. TR: performed the LC-MS/MS analysis and helped draft the manuscript. JJ: performed the LC-MS/MS analysis of certain Anabaena strains. LR: revised the manuscript. KS: participated in coordination of the study at HU and revised the manuscript. TK and KSJ participated in the design of the study, interpretation of the results and revision of the manuscript. All authors read and approved the final manuscript.

## Supplementary Material

Additional file 1 Phylogenetic analysis of adenylation domain amino acid sequences including B-type of McyB1 sequences from *Microcystis*. Click here for file
