# Supplementary material for: Evidence for positive selection acting on microcystin synthetase adenylation domains in three cyanobacterial genera
Source: BMC Evol Biol. 2008 Sep 22;8:256. doi: 10.1186/1471-2148-8-256 (PMC2564945; doi:10.1186/1471-2148-8-256)
Supplement: Additional file 1 — Phylogenetic analysis of adenylation domain amino acid sequences including B-type of McyB1 sequences from Microcystis. [file 1471-2148-8-256-S1.doc]

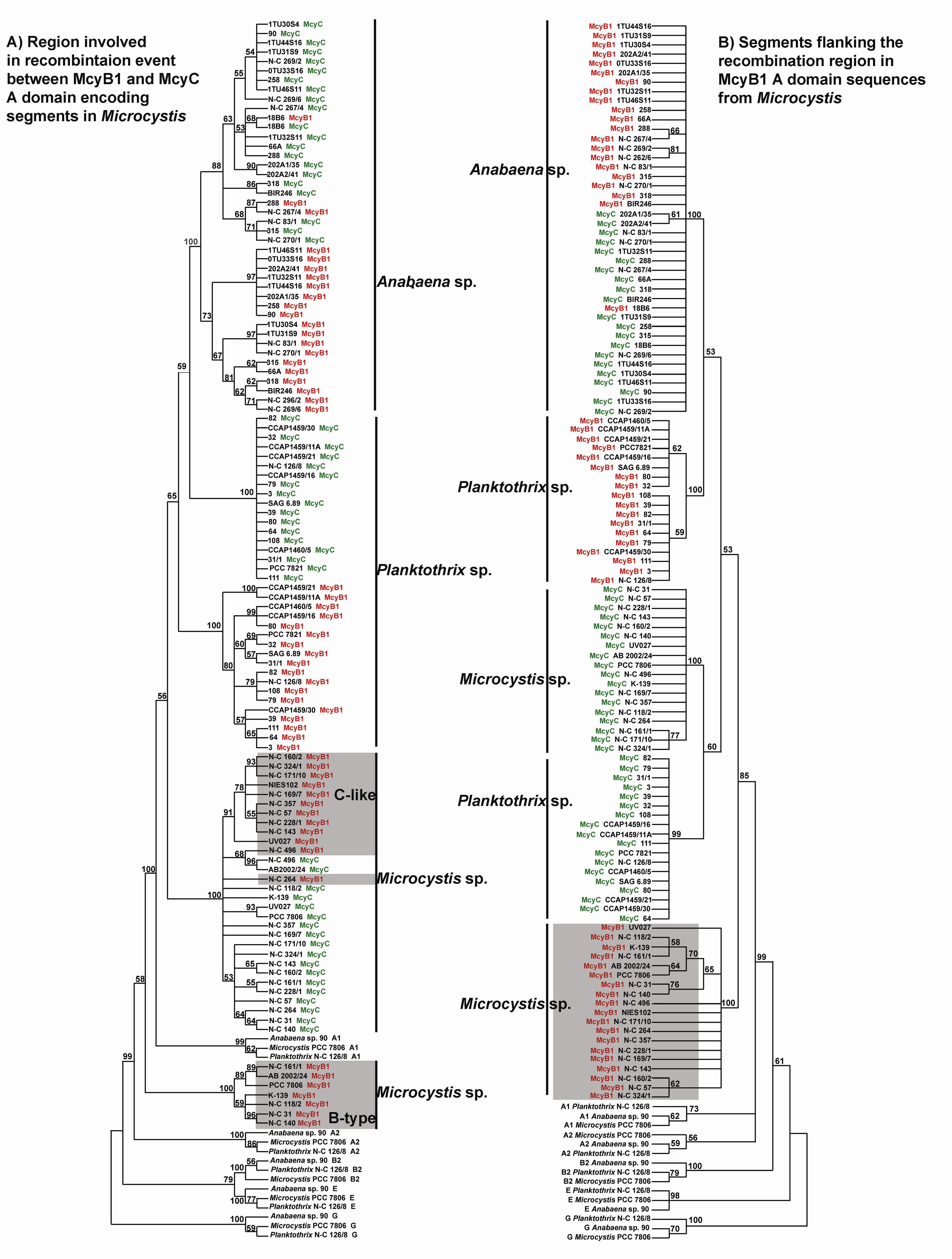


**Figure S1.** Phylogenetic analysis of adenylation domain amino acid sequences. Condensed neighbour-joining tree is shown, with bootstrap values above 50%. The tree was produced using Mega3 [1] under default conditions.

1. Phylogenetic analysis of region of A domains involved in recombination event between *mcyB* and *mcyC* segments encoding A-domains in *Microcystis*.
2. Phylogenetic analysis of segments flanking the recombination region in *mcyB* of *Microcystis*.

Reference:

1. Kumar S, Tamura K, Nei M**: MEGA3: Integrated Software for Molecular Evolutionary Genetics Analysis and Sequence Alignmen***t Briefings in Bioinformatic*s 2004**,** 5:150-163.
